# Supplementary material for: Cross sectional study of Twitter (X) use among academic anesthesiology departments in the United States
Source: PLoS One. 2024 Feb 8;19(2):e0298741. doi: 10.1371/journal.pone.0298741 (PMC10852312; doi:10.1371/journal.pone.0298741)
Supplement: Attachment — Submitted filename: Response to reviewers.docx [file pone.0298741.s002.docx]

Journal Requirements:

1. When submitting your revision, we need you to address these additional requirements.

Please ensure that your manuscript meets PLOS ONE's style requirements, including those for file naming. The PLOS ONE style templates can be found at 
https://journals.plos.org/plosone/s/file?id=wjVg/PLOSOne_formatting_sample_main_body.pdf and 
<https://journals.plos.org/plosone/s/file?id=ba62/PLOSOne_formatting_sample_title_authors_affiliations.pdf>

**Response: We have updated all style requirements based on the two links above.**

2. Did you know that depositing data in a repository is associated with up to a 25% citation advantage (https://doi.org/10.1371/journal.pone.0230416)? If you’ve not already done so, consider depositing your raw data in a repository to ensure your work is read, appreciated and cited by the largest possible audience. You’ll also earn an Accessible Data icon on your published paper if you deposit your data in any participating repository (<https://plos.org/open-science/open-data/#accessible-data>).

**Response: We have deposited the analytic dataset in this submission as a supporting information file.**

3. In your Methods section, please include additional information about your dataset and ensure that you have included a statement specifying whether the collection and analysis method complied with the terms and conditions for the source of the data.

**Response: We have added this into the manuscript.**

4. In your Data Availability statement, you have not specified where the minimal data set underlying the results described in your manuscript can be found. PLOS defines a study's minimal data set as the underlying data used to reach the conclusions drawn in the manuscript and any additional data required to replicate the reported study findings in their entirety. All PLOS journals require that the minimal data set be made fully available. For more information about our data policy, please see http://journals.plos.org/plosone/s/data-availability.

Upon re-submitting your revised manuscript, please upload your study’s minimal underlying data set as either Supporting Information files or to a stable, public repository and include the relevant URLs, DOIs, or accession numbers within your revised cover letter. For a list of acceptable repositories, please see http://journals.plos.org/plosone/s/data-availability#loc-recommended-repositories. Any potentially identifying patient information must be fully anonymized.

Important: If there are ethical or legal restrictions to sharing your data publicly, please explain these restrictions in detail. Please see our guidelines for more information on what we consider unacceptable restrictions to publicly sharing data: http://journals.plos.org/plosone/s/data-availability#loc-unacceptable-data-access-restrictions. Note that it is not acceptable for the authors to be the sole named individuals responsible for ensuring data access.

We will update your Data Availability statement to reflect the information you provide in your cover letter.

**Response: We have made the analytic dataset used for this study available as a supporting information file. There are no HIPAA related or privacy issues, as this study was non-human subjects’ research.**

5. We note that you have stated that you will provide repository information for your data at acceptance. Should your manuscript be accepted for publication, we will hold it until you provide the relevant accession numbers or DOIs necessary to access your data. If you wish to make changes to your Data Availability statement, please describe these changes in your cover letter and we will update your Data Availability statement to reflect the information you provide.

**Response: We are making the analytic dataset available as a supporting information file. The manuscript has been updated to reflect this.**

6. Please include captions for your Supporting Information files at the end of your manuscript, and update any in-text citations to match accordingly. Please see our Supporting Information guidelines for more information: http://journals.plos.org/plosone/s/supporting-information.

**Response: This has been done.**

7. Please review your reference list to ensure that it is complete and correct. If you have cited papers that have been retracted, please include the rationale for doing so in the manuscript text, or remove these references and replace them with relevant current references. Any changes to the reference list should be mentioned in the rebuttal letter that accompanies your revised manuscript. If you need to cite a retracted article, indicate the article’s retracted status in the References list and also include a citation and full reference for the retraction notice.

**Response: We have double checked all references for correctness.**

[Note: HTML markup is below. Please do not edit.]

Reviewers' comments:

Reviewer's Responses to Questions

**Comments to the Author**

1. Is the manuscript technically sound, and do the data support the conclusions?

The manuscript must describe a technically sound piece of scientific research with data that supports the conclusions. Experiments must have been conducted rigorously, with appropriate controls, replication, and sample sizes. The conclusions must be drawn appropriately based on the data presented.

Reviewer #1: Partly

Reviewer #2: Yes

**Response: No change required.**

2. Has the statistical analysis been performed appropriately and rigorously?

Reviewer #1: Yes

Reviewer #2: N/A

**Response: No change required.**

3. Have the authors made all data underlying the findings in their manuscript fully available?

The [PLOS Data policy](https://na01.safelinks.protection.outlook.com/?url=http%3A%2F%2Fwww.plosone.org%2Fstatic%2Fpolicies.action%23sharing&data=05%7C02%7C%7C0880b0a885ff4dfe2f5208dbfb6653c8%7C84df9e7fe9f640afb435aaaaaaaaaaaa%7C1%7C0%7C638380189140630317%7CUnknown%7CTWFpbGZsb3d8eyJWIjoiMC4wLjAwMDAiLCJQIjoiV2luMzIiLCJBTiI6Ik1haWwiLCJXVCI6Mn0%3D%7C3000%7C%7C%7C&sdata=VHEp4QnajMbfquQKPpDekkO3WBZqE8aZp1iTnhu53eE%3D&reserved=0) requires authors to make all data underlying the findings described in their manuscript fully available without restriction, with rare exception (please refer to the Data Availability Statement in the manuscript PDF file). The data should be provided as part of the manuscript or its supporting information, or deposited to a public repository. For example, in addition to summary statistics, the data points behind means, medians and variance measures should be available. If there are restrictions on publicly sharing data—e.g. participant privacy or use of data from a third party—those must be specified.

Reviewer #1: Yes

Reviewer #2: Yes

**Response: The analytic dataset has been made available as a supporting information file.**

4. Is the manuscript presented in an intelligible fashion and written in standard English?

PLOS ONE does not copyedit accepted manuscripts, so the language in submitted articles must be clear, correct, and unambiguous. Any typographical or grammatical errors should be corrected at revision, so please note any specific errors here.

Reviewer #1: Yes

Reviewer #2: Yes

**Response: No change required.**

5. Review Comments to the Author

Please use the space provided to explain your answers to the questions above. You may also include additional comments for the author, including concerns about dual publication, research ethics, or publication ethics. (Please upload your review as an attachment if it exceeds 20,000 characters)

Reviewer #1: Your paper has been written focusing on Anesthesiology which explains why your references are limited. I suggest you broaden the search and compare with other medical and dental specialties for better comparison.

**Response: We have extensively updated our discussion to be more comprehensive. We now talk about the use of social media, mostly Twitter (X) by surgeons, ER physicians, dentists, and nurses.**

Reviewer #2: The author researched the utilization of Twitter (X) in anethsology. That was interesting and fit to the current world perseption. Let me ask one question.

/Do you have any information about other platform of social media

**Response: No we only collected data from Twitter, which was itself a VERY large undertaking. We have added this as a limitation of the study in the Discussion section.**

/What did this research academically mean?

**Response: This is a fair question. Overall one of our major points is that social media has enormous potential to disseminate information, enhance collaboration, and provide education to both professionals and the public. At the present time most academic anesthesiology departments are primarily using Twitter (X) to promote their own departments and faculty and disseminate news. There are opportunities in our opinion to use the platform for other purposes including providing education to the public about what physician anesthesiologists do on a day-to-day basis. Also, Twitter (X) is a powerful tool to disseminate important research findings, which could be done more by academic anesthesiology departments.**

**We have added content related to this into the Discussion.**

6. PLOS authors have the option to publish the peer review history of their article ([what does this mean?](https://na01.safelinks.protection.outlook.com/?url=https%3A%2F%2Fjournals.plos.org%2Fplosone%2Fs%2Feditorial-and-peer-review-process%23loc-peer-review-history&data=05%7C02%7C%7C0880b0a885ff4dfe2f5208dbfb6653c8%7C84df9e7fe9f640afb435aaaaaaaaaaaa%7C1%7C0%7C638380189140630317%7CUnknown%7CTWFpbGZsb3d8eyJWIjoiMC4wLjAwMDAiLCJQIjoiV2luMzIiLCJBTiI6Ik1haWwiLCJXVCI6Mn0%3D%7C3000%7C%7C%7C&sdata=Dm%2FFaOm0KV7OihfwW0vnVmbDEk3XG5xlY3u%2B4KL8Noo%3D&reserved=0)). If published, this will include your full peer review and any attached files.


If you choose “no”, your identity will remain anonymous but your review may still be made public.


**Do you want your identity to be public for this peer review?** For information about this choice, including consent withdrawal, please see our [Privacy Policy](https://na01.safelinks.protection.outlook.com/?url=https%3A%2F%2Fwww.plos.org%2Fprivacy-policy&data=05%7C02%7C%7C0880b0a885ff4dfe2f5208dbfb6653c8%7C84df9e7fe9f640afb435aaaaaaaaaaaa%7C1%7C0%7C638380189140630317%7CUnknown%7CTWFpbGZsb3d8eyJWIjoiMC4wLjAwMDAiLCJQIjoiV2luMzIiLCJBTiI6Ik1haWwiLCJXVCI6Mn0%3D%7C3000%7C%7C%7C&sdata=nre0ls%2FFR0lhBka2X%2BjgJX6vZT0oSS%2BsSjRFEFHgT%2Bg%3D&reserved=0).

Reviewer #1: No

Reviewer #2: No

**Response: No change required.**
